# Radiative Neutron β-Decay in Effective Field Theory

**DOI:** 10.6028/jres.110.062

**Published:** 2005-08-01

**Authors:** Susan Gardner, Véronique Bernard, Ulf-G. Meißner, Chi Zhang

**Affiliations:** Department of Physics and Astronomy, University of Kentucky, Lexington, Kentucky 40506-0055, USA; Université Louis Pasteur, Laboratoire de Physique Théorique 3–5, rue de l’Université, F-67084 Strasbourg, France; Universität Bonn, Helmholtz-Institut für Strahlen-und Kernphysik (Theorie) Nußallee 14–16, D-53115 Bonn, GermanyForschungszentrum Jülich, Institut für Kernphysik (Theorie) D-52425 Jülich, Germany; Department of Physics and Astronomy, University of Kentucky, Lexington, Kentucky 40506-0055, USA

**Keywords:** neutron *β*-decay, radiative corrections

## Abstract

We consider radiative *β*-decay of the neutron in heavy baryon chiral perturbation theory. Nucleon-structure effects not encoded in the weak coupling constants *g_A_* and *g_V_* are determined at next-to-leading order in the chiral expansion, and enter at the O(0.5%)-level, making a sensitive test of the Dirac structure of the weak currents possible.

## 1. Framework

Experimental studies of *β*-decay at low energies have played a crucial role in the rise of the Standard Model (SM) [1]. In recent years, continuing, precision studies of neutron *β*-decay have been performed, to better both the determination of the neutron lifetime and of the correlation coefficients. To realize a SM test to a precision of ≈1 % or better requires the application of radiative corrections [2]. One component of such, the “outer” radiative correction, is captured by electromagnetic interactions with the charged, final-state particles, in the limit in which their structure is neglected. In this, neutron radiative *β*-decay enters, and we consider it explicitly. We do so in part (i) to study the hadron matrix elements in O(1/M), as the same matrix elements, albeit at different momentum transfers, enter in muon radiative capture [3], and (ii) to test the Dirac structure of the weak current, through the determination of the circular polarization of the associated photon [4, 5]. Here we report on our recent work—please see Ref. [6] for all details.

In neutron radiative *β*-decay, bremsstrahlung from either charged particle can occur, and radiation can be emitted from the effective weak vertex. In the pioneering work of Ref. [4] only the bremsstrahlung terms are computed—this suffices only if all O(1/M) terms are neglected. Here we describe a systematic analysis of neutron radiative *β*-decay in the framework of heavy baryon chiral perturbation theory (HBCHPT) [7, 8, 9] and in the small scale expansion (SSE) [10], including all terms in O(1/M), i.e., at next-to-leading order (NLO) in the small parameter *ε* [6]. We note that *ε* collects all the small external momenta and quark (pion) masses, relative to the heavy baryon mass *M*, which appear when HBCHPT is utilized; in case of the SSE, such is supplemented by the Δ(1232)-nucleon mass splitting, relative to *M*, as well. These systematic approaches allow us to calculate the recoil-order corrections in a controlled way.

We consider $n(p)\to p(p^{\prime })+e^{-}(l_{e})+{\bar{v}}_{e}(l_{v})+\gamma (k)$, where *p, p*′, *l*_e_, *l*_ν_, and *k* denote the four-momentum of the neutron, proton, electron, anti-neutrino, and photon, respectively—we denote the photon energy by *ω.* At low energies, the matrix element for radiative neutron *β*-decay decomposes into two pieces,

$$\begin{array}{l} ℳ(n\to pe^{-}{\bar{v}}_{e}\gamma )\\ \;=i\frac{g^{\alpha \beta }}{M_{W}^{2}}[\langle {\bar{v}}_{e}e^{-}|J_{\alpha }^{-}|0\rangle \langle p|T(V\cdot \varepsilon *V_{\beta }^{+}-V\cdot \varepsilon *A_{\beta }^{+})|n\rangle \\ \;+\langle {\bar{v}}_{e}e^{-}\gamma |J_{\alpha }^{-}|0\rangle \langle p|V_{\beta }^{+}-A_{\beta }^{+}|n\rangle ], \end{array}$$ (1)

in terms of the leptonic weak current (*J*^−^), as well as the hadronic vector (*V*) and axial.vector (*A*) currents. Note that *ε*_µ_ is the photon polarization vector and *M*_W_ is the W-boson mass. The first term includes bremsstrahlung from the proton, as well as radiation from the effective weak vertex, whereas the second term describes bremsstrahlung from the electron. We now turn to the leptonic and hadronic matrix elements which appear. The leptonic current matrix elements follow from QED, in concert with the V-A structure of the weak current. The latter, cum Lorentz and translational invariance [11], also fixes $\langle p|V_{v}^{+}-A_{v}^{+}|n\rangle$; the form factors which appear therein can be determined from experiment. To compute the remaining matrix elements, $\langle p|T(V\cdot \varepsilon *V_{v}^{+}-V\cdot \varepsilon^{*}A_{v}^{+})|n\rangle$, we employ HBCHPT. Thus the heavy baryon is treated non-relativistically, and its interactions are organized in powers of *ε*. We work in O(1/M) throughout, so that our matrix elements include photon emission from the weak vertex as well. For consistency we also treat $\langle p|V_{v}^{+}-A_{v}^{+}|n\rangle$ in the non-relativistic limit, expanding to $O(1/M^{2})$ throughout. We note that the pertinent two- and four-point functions can be taken directly from Ref. [3], after relabeling the momenta and such [6]. Working in the Coulomb gauge ε*·υ = 0 for the photon and making use of the transversality condition ε*·k = 0, we find $\langle p|T(V\cdot \varepsilon *V_{\nu }^{+}-V\cdot \varepsilon *A_{v}^{+})|n\rangle$ is of O(1/M), so that only electron bremsstrahlung makes an O(1) contribution to radiative neutron *β*-decay.

## 2. Results

**Fig. 1:**
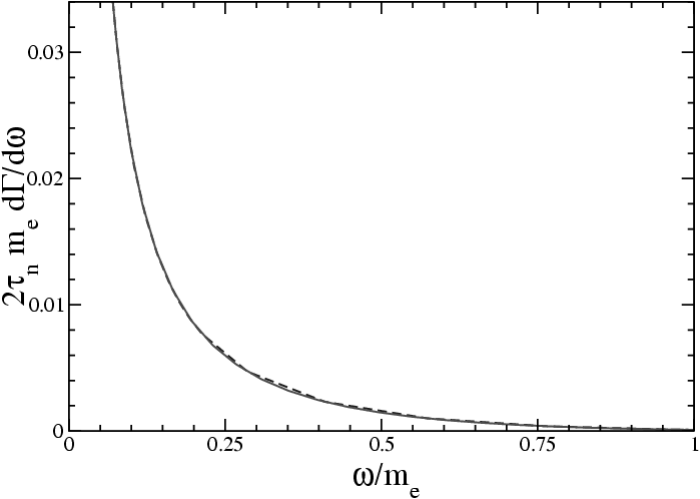
The photon energy spectrum for radiative neutron *β*-decay. The dashed line denotes the result to NLO in the SSE, whereas the solid line denotes the leading order result, employed in Ref. [4].

We now present our results [6]. We show the photon energy spectrum dΓ/d*ω* in Fig. 1, and for the total branching ratio, which depends on the range chosen for *ω*, we find,

$$\begin{array}{r} \omega \in [0.005\;\text{MeV},\;0.035\;\text{MeV}],\;\text{Br}:2.59\cdot 10^{-3},\\ \omega \in [0.035\;\text{MeV,}\;0.100\;\text{MeV}],\;\text{Br}:1.11\cdot 10^{-3},\\ \omega \in [0.100\;\text{MeV},\;\omega^{\max}=0.782\;\text{MeV}],\;\text{Br}:0.72\cdot 10^{-3}. \end{array}$$ (2)

The branching ratio determined for *ω* ∈ [0.035 MeV, 0.100 MeV] can be compared directly with the experimental limit of Br < 6.9·10^−3^ (90 % CL) [12], with which it is compatible. In Fig. 1 we superimpose the numerical results we find with those using the leading order form of $\sum_{\text{spins}}|ℳ{|}^{2}$. The two curves can scarcely be distinguished; indeed, the recoil-order corrections to the matrix elements are no larger than O(0.5%). The SSE contribution is itself of  O(0.1%). In contrast, the recoil-order corrections to the *A* and *a* correlations in neutron *β*-decay are of O(1−2%) [13]; apparently, the appearance of an additional particle in the final state makes the recoil-order corrections smaller still.

We also compute the polarization of the emitted photon. Defining the polarization states such that *ε_L_*, e.g., does indeed correspond to a left-handed photon when ***k***║***l***_e_ [6], we determine the polarization *P* via *P* = (Γ*_R_ −* Γ*_L_*)/(Γ*_R_−Γ_L_*). We can also study the polarization as a function of *ω* and *E_e_* as well; in such cases, we define *P* (*ω*) by replacing Γ*_L,R_* with *d*Γ*_L,R_/dω* and *P*(*ω*,*E*_e_) by replacing Γ*_L,R_* with *d*^2^Γ*_L,R_/dωdE*_e_. We find that the polarization evolves from near-zero at low photon energies to nearly 100 % left-handed polarization at high photon energies, as consistent with the discussion of Ref. [5].

**Fig. 2:**
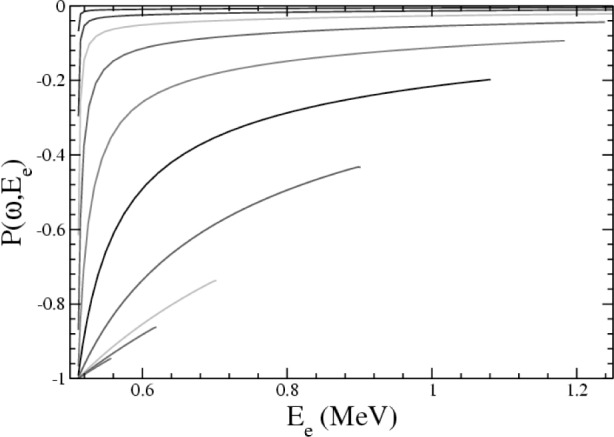
The photon polarization *P*(*ω, E*_e_) in radiative neutron *β*-decay to NLO in the SSE, as a function of *E_e_* for $(E_{e}^{\max}-E_{e})/E_{e}^{\max}≳0.2\%$ and various, fixed *ω*. For *E*_e_ such that $(E_{e}^{\max}-E_{e})/E_{e}^{\max}≲0.2\%$, the polarization plunges to −1, see text. The curves from smallest absolute polarization to largest have *ω* = 0.00539, 0.0135, 0.0265, 0.0534, 0.109, 0.209, 0.390, 0.589, 0.673, and 0.736 MeV, respectively.

The evolution of the polarization with *ω* is dissected in Fig. 2; as *ω* grows large, the associated electron momentum is pushed towards zero, and the absolute polarization grows larger. This follows as in the circular basis we can replace (2ε±*⋅le−kε±*) in $\langle {\bar{v}}_{e}e^{-}\gamma |J_{\mu }^{-}|0\rangle$ with (2ε±*⋅le−ω(1±γ5)γ0ε±*) with $\varepsilon_{+,-}=\varepsilon_{R,L}$. The photon associated with the first term has no circular polarization; this contribution vanishes if |***l***_e_| = 0. In this observable as well the O(1/M) contributions are O(0.5%) or less. Interestingly, the inclusion of these contributions does not impact the determined polarization to an appreciable degree when ***l***_e_║ ± ***k***; *P* ≈ −1. Note that as *E_e_* approaches $E_{e}^{\max}(\omega )$, ***l***_e_ becomes parallel to −***k***, so that ε*·*l*_e_ approaches zero and *P* approaches −1 to a high degree of accuracy. In neutron radiative *β*-decay, the polarization can differ appreciably from unity, so that the calculation of the polarization is *necessary* to realize a SM test; significant deviations from this prediction would nevertheless signify the palpable presence of a left-handed anti-neutrino or of non-*V-A* currents. As noted by Martin and Glauber [5], the polarization of the photon in *S*-state orbital electron capture is also sensitive to the *phase* of the vector and axial-vector couplings in the low-energy interaction Hamiltonian [14] if the anti-neutrino is no longer assumed to be strictly right-handed. Such expectations apply to neutron radiative *β*-decay as well, so that the photon polarization can probe new physics effects to which the correlation coefficients in neutron *β*-decay are insensitive [15].

In summary, we have computed the photon energy spectrum and photon polarization in neutron radiative *β*-decay in an effective field theory approach utilizing HBCHPT and the SSE, including all terms in O(1/M). The leading contribution to the photon energy spectrum has been calculated previously [4]; we agree with the expression in Ref. [4] for $\sum_{\text{spins}}|ℳ{|}^{2}$, though we disagree with their numerical results for the photon energy spectrum. Moreover, we .nd that the O(1/M) terms are numerically quite small, generating contributions no larger than O(0.5%), so that radiative neutron *β*-decay is quite insensitive to nucleon structure effects beyond those encoded in *g_V_* and *g_A_.* We have found that nucleon structure effects have a similarly negligible role in the determination of the photon polarization, so that a precise measurement of the photon polarization may well offer a crisp diagnostic of non-SM effects.

## References

[1] Dubbers D (1999). Nucl Phys A.

[2] Marciano WJ, Sirlin A (1986). Phys Rev Lett.

[3] Bernard V, Hemmert TR, Meißner U-G (2001). Nucl Phys A.

[4] Gaponov YV, Khafizov RU (1996). Phys Atom Nucl.

[5] Martin PC, Glauber RJ (1958). Phys Rev.

[6] Bernard V, Gardner S, Meißner U-G, Zhang C (2004). Phys Lett B.

[7] Jenkins E, Manohar AV (1991). Phys Lett B.

[8] Bernard V, Kaiser N, Kambor J, Meißner U-G (1992). Nucl Phys B.

[9] Bernard V, Kaiser N, Meißner U-G (1995). Int J Mod Phys E.

[10] Hemmert TR, Holstein BR, Kambor J (1998). J Phys G.

[11] Goldberger ML, Trieman SB (1958). Phys Rev.

[12] Beck M (2002). JETP Lett.

[13] Gardner S, Zhang C (2001). Phys Rev Lett.

[14] Lee TD, Yang CN (1956). Phys Rev.

[15] Jackson JD, Treiman SB, Wyld HW (1957). Phys Rev.

